# Supplementary material for: MetaQTL: a package of new computational methods for the meta-analysis of QTL mapping experiments
Source: BMC Bioinformatics. 2007 Feb 8;8:49. doi: 10.1186/1471-2105-8-49 (PMC1808479; doi:10.1186/1471-2105-8-49)
Supplement: Additional File 7 — MetaQTL Package : jar file and tutorial. This Zip archive contains both the MetaQTL JAR file and the files of the tutorial. [file 1471-2105-8-49-S7.zip › org.inra.metaqtl/doc/org/metaqtl/package-use.html]

Uses of Package org.metaqtl


|  |  |  |
| --- | --- | --- |
| **Overview** \| **Package** \| Class \| **Use** \| **Tree** \| **Deprecated** \| **Index** \| **Help** | |  |
| PREV   NEXT | **FRAMES**    **NO FRAMES**     **All Classes** |  |


---


## **Uses of Package org.metaqtl**

| Packages that use org.metaqtl | |
| --- | --- |
| **org.metaqtl** |  |
| **org.metaqtl.adapter** |  |
| **org.metaqtl.algo** |  |
| **org.metaqtl.factory** |  |
| **org.metaqtl.graph** |  |
| **org.metaqtl.main** |  |

Classes in org.metaqtl used by org.metaqtl

****ChromCluster****
  
          A cluster of chromosomes which share the same name inside a
`MapCluster` framwork.

****ChromCluster.ClusterGraph****

****Chromosome****
  
          On a Chromosome there are two types of locus :
Marker
QTL (Quantitative Trait Loci)
inside a mapping context which is defined by both the mapping framework
(function and unit) and the population cross design used to build this
chromosome.

****CMarkerSequence****
  
          A Common marker sequence between two ordered set of markers
is a sub-set of at least two markers which order is the same between
the two sets (may be reverse).

****EMCriteria****

****EMResult****

****MetaChrom****
  
          A MetaChrom is the result of the merge of the members of a
`ChromCluster`.

****MetaDico****
  
          This class represents a dictionnary of synonyms.

****MetaQtlModel****

****MetaQtlResult****
  
          A `MetaQtlResult` is used to store the
results of a QTL meta analysis which has been performed
for a given trait group on a given chromosome.

****MultiFitResult****
  
          This class represents a result of a multilinear regression
analysis.

****Qtl****
  
          A QTL (Quantitative Trait Loci)

****Qtl.QTLCross****
  
          This class stores the informations on the cross
design used to detect the QTL.

****Qtl.QTLProj****
  
          This class stores the QTL projection
properties.

****Qtl.QTLStats****
  
          This class stores the usual statistics for the QTL.

****Qtl.QTLSupport****
  
          This class stores information
on the QTL Confidence Interval

****Qtl.QTLTrait****
  
          This class stores the trait description of a QTL

****Tree****
  
          This class defines a tree.

****TreeNode****
  
          This class represents a node in a binary Tree obtained
when applying usual hirarchical clustering methods.

Classes in org.metaqtl used by org.metaqtl.adapter

****Chromosome****
  
          On a Chromosome there are two types of locus :
Marker
QTL (Quantitative Trait Loci)
inside a mapping context which is defined by both the mapping framework
(function and unit) and the population cross design used to build this
chromosome.

****EMResult****

****MetaMap****
  
          A `MetaMap` is a bundle of `MetaChrom` objects.

****MetaQtlAnalysis****
  
          A QTL meta analysis consists in a clustering of QTL positions over
chromosomes for different group of traits.

****MetaQtlData****
  
          QTL meta analysis first requires to gather the QTL according
to the group trait they belong and then to format the QTL data
into data points to perfom clustering.

****MetaQtlResult****
  
          A `MetaQtlResult` is used to store the
results of a QTL meta analysis which has been performed
for a given trait group on a given chromosome.

****Qtl****
  
          A QTL (Quantitative Trait Loci)

****QtlPartition****

Classes in org.metaqtl used by org.metaqtl.algo

****ChromCluster****
  
          A cluster of chromosomes which share the same name inside a
`MapCluster` framwork.

****Chromosome****
  
          On a Chromosome there are two types of locus :
Marker
QTL (Quantitative Trait Loci)
inside a mapping context which is defined by both the mapping framework
(function and unit) and the population cross design used to build this
chromosome.

****CMarkerSequence****
  
          A Common marker sequence between two ordered set of markers
is a sub-set of at least two markers which order is the same between
the two sets (may be reverse).

****EMResult****

****IMetaAlgorithm****
  
          This interface defines some methods which are common
to data analysis algorithms.

****MetaChrom****
  
          A MetaChrom is the result of the merge of the members of a
`ChromCluster`.

****MetaDico****
  
          This class represents a dictionnary of synonyms.

****MetaMap****
  
          A `MetaMap` is a bundle of `MetaChrom` objects.

****MetaQtlAnalysis****
  
          A QTL meta analysis consists in a clustering of QTL positions over
chromosomes for different group of traits.

****MetaQtlData****
  
          QTL meta analysis first requires to gather the QTL according
to the group trait they belong and then to format the QTL data
into data points to perfom clustering.

****MetaQtlResult****
  
          A `MetaQtlResult` is used to store the
results of a QTL meta analysis which has been performed
for a given trait group on a given chromosome.

****MultiFitResult****
  
          This class represents a result of a multilinear regression
analysis.

****Tree****
  
          This class defines a tree.

Classes in org.metaqtl used by org.metaqtl.factory

****EMResult****

****MetaDico****
  
          This class represents a dictionnary of synonyms.

****MetaMap****
  
          A `MetaMap` is a bundle of `MetaChrom` objects.

****MetaQtlAnalysis****
  
          A QTL meta analysis consists in a clustering of QTL positions over
chromosomes for different group of traits.

****MetaQtlModel****

****Tree****
  
          This class defines a tree.

Classes in org.metaqtl used by org.metaqtl.graph

****Chromosome****
  
          On a Chromosome there are two types of locus :
Marker
QTL (Quantitative Trait Loci)
inside a mapping context which is defined by both the mapping framework
(function and unit) and the population cross design used to build this
chromosome.

****QtlPartition****

Classes in org.metaqtl used by org.metaqtl.main

****ChromCluster****
  
          A cluster of chromosomes which share the same name inside a
`MapCluster` framwork.

****Chromosome****
  
          On a Chromosome there are two types of locus :
Marker
QTL (Quantitative Trait Loci)
inside a mapping context which is defined by both the mapping framework
(function and unit) and the population cross design used to build this
chromosome.

****MetaDico****
  
          This class represents a dictionnary of synonyms.

****MetaQtlAnalysis****
  
          A QTL meta analysis consists in a clustering of QTL positions over
chromosomes for different group of traits.

****MetaQtlModel****

****QtlPartition****

---


|  |  |  |
| --- | --- | --- |
| **Overview** \| **Package** \| Class \| **Use** \| **Tree** \| **Deprecated** \| **Index** \| **Help** | |  |
| PREV   NEXT | **FRAMES**    **NO FRAMES**     **All Classes** |  |


---
